# Gene editing for collagen disorders: current advances and future perspectives

**DOI:** 10.1038/s41434-025-00560-7

**Published:** 2025-08-11

**Authors:** Klaudia Kocsy, Harry Wilkinson, Favour Felix-Ilemhenbhio, Benjamin Bax, Tom Van Agtmael, Mimoun Azzouz, Arshad Majid

**Affiliations:** 1 School of Medicine and Population Health, Sheffield Institute for Translational Neuroscience (SITraN), University of Sheffield, Sheffield, UK; 2 Medicines Discovery Institute, Cardiff University, Cardiff, UK; 3 School of Cardiovascular & Metabolic Health, University of Glasgow, Glasgow, UK

**Keywords:** RNAi, Targeted gene repair

## Abstract

Collagen disorders encompass a wide range of genetic conditions caused by pathogenic variants in collagen genes for which there is an unmet need for treatments. They present various clinical features, ranging from localised tissue abnormalities to severe systemic complications. Symptoms differ significantly and depend on the pathogenic variant, which can affect various systems, including the musculoskeletal, cardiovascular, and respiratory systems, highlighting the complex implications of collagen gene pathogenic variants and the wide range of expression patterns among different collagen types. Gene-editing technologies, particularly Clustered Regularly Interspaced Palindromic Repeats (CRISPR)-Cas systems, have emerged as promising therapeutic options for these disorders, representing a putative one-for-all treatment strategy. This review provides an overview of current gene-editing strategies aimed at collagen-related diseases, including osteogenesis imperfecta, Alport syndrome, and dystrophic epidermolysis bullosa. We explore the application of CRISPR-Cas9, which facilitates targeted DNA modifications, base editing (BE), and prime editing (PE), enabling precise single-nucleotide alterations without double-strand breaks (DSB). Preclinical and clinical studies have shown the potential of gene therapy to enhance collagen production, restore tissue integrity, and alleviate symptoms. However, challenges persist, including the lack of recurring mutations, the need for improved delivery methods, the reduction of off-target effects, and the development of novel therapies. Despite these challenges, advancements in gene editing techniques appear promising in enhancing editing efficiency while minimising unintended mutations, paving the way for more precise and safer genetic interventions for collagen disorders. Gene editing is fundamentally transforming medicine and biotechnology. Its applications encompass advanced diagnostics, tailored therapeutic strategies, and solutions for rare genetic disorders. By enabling precise genetic modifications, gene editing is paving the way for treatments of previously untreatable diseases, including those linked to collagen pathogenic variants. This review discusses the latest advancements in gene therapy techniques targeting collagen-related disorders. It explores innovative approaches like CRISPR-Cas9-mediated gene editing and highlights emerging strategies, such as allele-specific inactivation and base editing (BE). By examining these cutting-edge therapies and their potential clinical applications, this review highlights the transformative impact of gene editing in treating collagen-related conditions, while also identifying critical challenges and future directions for research.

## Overview of Collagen

Collagen is the most abundant protein in both vertebrates and invertebrates, playing a crucial role in maintaining structural integrity and facilitating numerous physiological processes [1]. Traditionally, it was believed that collagen constituted approximately 30% of the total protein in mammals, including humans [1]. However, recent studies have challenged this long-held assumption [2]. A study on mice found that collagen makes up ~12% of total protein in females and 17% in males, and collagen content varies significantly between different tissues (0.1% in the brain and liver, 1% in the heart and kidney, 4% in the muscle and lung, 20–40% in the skin, 25–35% in bones, and 40–50% in tendons of wild-type mice) [2].

**Fig. 1:**
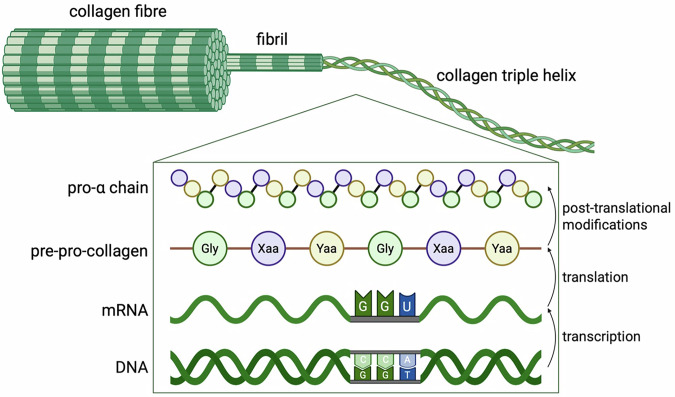
Fibrillar collagen primary structure. Collagen synthesis begins with the transcription of specific genes (e.g., COL1A1) in the nucleus. mRNA is synthesised and translated into pre-pro-collagen, which undergoes post-translational modifications to form the pro-α chains. Three collagen monomers bind, forming a right-handed triple helix. The collagen structure consists of Gly-Xaa-Yaa repeats, with glycine being critical at every third position to accommodate the tight helical structure. The Xaa and Yaa residues can be any amino acid, but are often proline and hydroxyproline. For fibrillar collagens like collagen I and III, the pro-collagen is cleaved and assembles into fibrils, which then combine to create larger fibres. These fibres play crucial roles throughout the body, providing structural support, strength, and flexibility to various tissues and organs. (Detailed review on collagen assembly [107]).

The 28 distinct types of collagen proteins (types I to XXVIII) in humans are encoded by at least 45 genes, which are collectively referred to as the collagen gene family. These genes, including *COL1A1*, *COL4A3*, and *COL7A1*, are characterised by their repetitive exon structure and conserved splice sites, reflecting the Gly-Xaa-Yaa triplet motif crucial for triple helix formation [3]. Post-translational modifications (for review see [4]), such as the hydroxylation, glycosylation and galactosylation of proline and lysine residues, are essential for stabilising the collagen triple helix and facilitating cross-linking between collagen fibrils [3, 5] **(**Fig. 1**)**. The different collagens form distinct supramolecular structures, which in part form the basis of their classification into different collagen types, including fibrillar, network-forming, transmembrane, and FACIT collagens (Fibril-Associated Collagens with Interrupted Triple helices) [6].

Collagen is the primary protein in the extracellular matrix (ECM) with numerous functions. For instance, fibrillar collagens provide tensile strength and maintain tissue integrity. In physiological processes, collagen is essential for wound healing, providing a scaffold for tissue regeneration and stimulating fibroblast proliferation and angiogenesis. Beyond wound healing, collagen supports the structure of organs and maintains blood vessel integrity, underscoring its critical role in overall health [7].

Each collagen type plays a specific role in maintaining the structure and function of particular tissues [1], and in humans, types I to V are the most common [8]. Type I predominates in skin, bone, and tendons, providing tensile strength. Type II, found in articular cartilage, provides shock absorption and elasticity. Types III and IV are present in blood vessels, where they provide structural support and elasticity; mutations in them cause genetic vascular diseases, such as Ehlers-Danlos Syndrome and Alport syndrome. Type IV is considered the major structural component of all basement membranes (BMs), which underlie endothelial and epithelial cells. Collagen IV is crucial for size and charge-selective filtration in the glomerular BMs of the kidneys [9], with *COL4A3-COL4A5* mutations leading to the genetic nephropathy (Alport Syndrome). Collagen VII forms anchoring fibrils, which are essential for the adhesion of the epidermis to the dermis; mutations in this protein cause Epidermolysis Bullosa [10]. Type V is present in the hair, corneas and some layers of the skin, where it regulates fibril assembly [9] with defects causing Ehlers-Danlos Syndrome.

At the cellular level, collagens affect cell function, including adhesion, migration, proliferation, and differentiation through specific signalling mechanisms. It interacts with integrins at particular binding sites, such as the GFOGER motif (Gly-Phe-Hyp-Gly-Glu-Arg) [11], which can trigger signalling pathways that influence cell behaviour. Some collagens, such as Collagen IV, can also bind to and induce discoidin domain receptor 1 (DDR1) and DDR2 tyrosine kinase transmembrane receptors, and induce their signalling [12], whereby DDR binding can strengthen integrin binding [13, 14]. Modulating cellular activities can also be achieved through the binding of G protein-coupled receptors. Additionally, proteolytic cleavage in the ECM can release biologically active peptides, known as matrikines, which influence angiogenesis and have been associated with diseases, such as cancer, pulmonary hypertension, heart failure with preserved ejection fraction, and osteoarthritis [15].

ECM composition and function, as well as the balance between collagen synthesis and degradation, are crucial and are maintained by a complex interplay of proteases and their inhibitors. Excessive collagen degradation by matrix metalloproteinases (MMPs), such as in chronic wounds or osteoarthritis, disrupts ECM homoeostasis and impairs tissue repair [16]. On the other hand, excessive collagen deposition drives desmoplasia, a pathological stromal reaction linked to tumour progression and metastatic dissemination through biomechanical and biochemical signalling pathways [17]. Beyond its structural role, collagen assembly and remodelling critically shape the tumour microenvironment (TME) by modulating immune cell infiltration, conferring therapy resistance, and influencing mechanical properties, such as matrix stiffness [18]. In solid tumours, aberrant cross-linking and alignment of collagen fibres generate a dense, rigid ECM that compromises the diffusion of gene-editing vectors, including viral particles and lipid nanoparticles, by creating steric hindrance and elevated interstitial pressure [19]. These structural barriers underscore the necessity of mapping ECM alterations to optimise the delivery and efficacy of targeted gene therapies, ensuring sufficient penetration to correct oncogenic mutations in neoplastic cells.

**Fig. 2:**
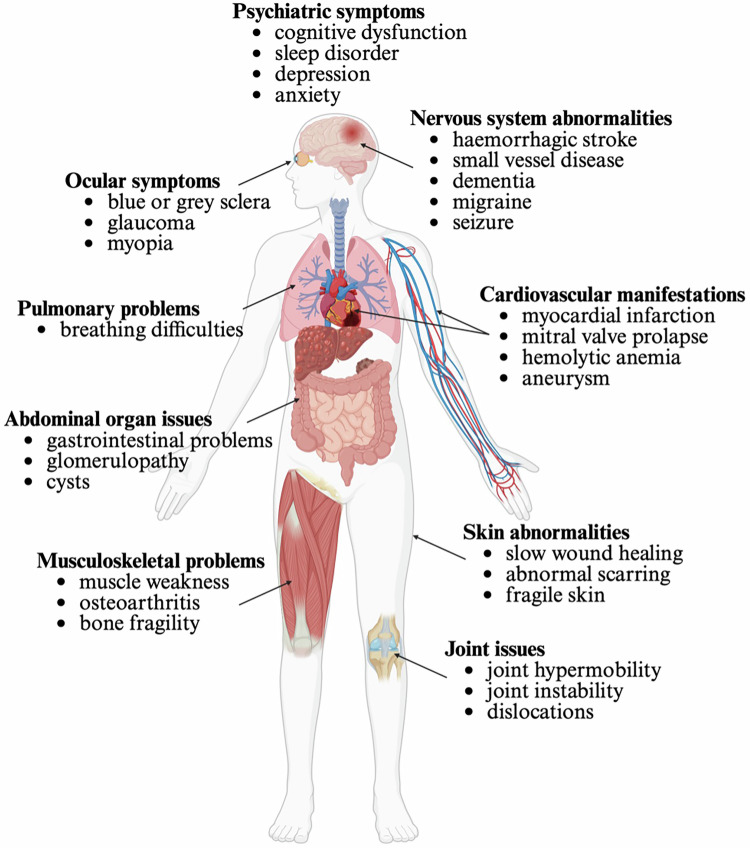
Clinical representations of collagen disorders. Schematic representation of the multi-systemic clinical features of various collagen disorders. Ocular symptoms include blue sclerae, glaucoma, and myopia. Neurological involvement can manifest as haemorrhagic stroke, small vessel disease, dementia, migraine, and seizures. Other common features include cardiovascular issues, pulmonary problems, abdominal organ issues, musculoskeletal problems, skin abnormalities and joint issues.

Pathogenic variants that disrupt the crucial triple-helical structure or interfere with post-translational modifications can affect collagen structure, stability, levels, and interactions with cell surface receptors [3], and lead to a variety of collagen diseases, ranging from mild to severe or even lethal (Fig. 2). Given the importance of the glycine residue in triple helix formation, it is unsurprising that the majority of missense mutations affect these residues [10]. These disorders manifest with a wide range of symptoms, including skin rashes, fatigue, muscle weakness, joint pain, fever, digestive issues, breathing difficulties, stroke, dementia, depression and organ-specific complications affecting the heart, lungs, and kidneys [20–22]. The correlation between genotype and phenotype is complex, with severity depending on the type and location of the pathogenic variant [20, 21]. Missense or nonsense mutations involving glycine substitution in the triple helix domain usually result in more severe manifestations due to their effect on supramolecular network formation and dominant negative effects [21, 22].

While collagen disorders have traditionally been considered ECM disorders, evidence from the past decade has implicated intracellular pathways in disease aetiology, for instance, including the activation of endoplasmic reticulum stress due to collagen misfolding [10, 23, 24] and effects on autophagy and mitochondrial function [25]. The combination of ECM-derived and intracellular responses and defects means that targeting downstream pathways and molecules in collagen disorders is problematic and will likely only rescue some aspects of the disorders, rather than the disease itself.

The collagen genes and their related disorders highlight the diversity of collagen’s biological roles and the broad range of diseases caused by their pathogenic variants. Over 1000 known pathogenic variants in collagen genes can lead to various genetic collagen disorders, affecting 17 out of the 28 types of collagen proteins [8]. However, to date, only one Food and Drug Administration (FDA)-approved gene therapy is available for collagen disorders, which targets *COL7A1* for the treatment of dystrophic epidermolysis bullosa [26]. This emphasises the need to explore potential interventions for other collagen-related disorders, providing hope for patients suffering from these debilitating conditions. The most common collagen disorders and their associated gene therapy approaches are summarised in Table 1.

**Table 1:** Common genetic disorders associated with collagen gene pathogenic variants.

Collagen Protein	Expressed by	Collagen Gene	Associated Disease	Gene Therapy	Ref
Type I (fibrillar)	osteoblasts, fibroblasts, mesenchymal cells	*COL1A1*	Ehlers-Danlos syndrome (classical/ vascular)		[40]
			Infantile cortical hyperostosis		[41]
			Osteogenesis imperfecta, types I–IV	CRISPR-Cas9-mediated HDR editing [36–39]	[35]
		*COL1A2*	Ehlers-Danlos syndrome (classical/ vascular)		[40]
			Ehlers-Danlos syndrome (cardiac-valvular)		[40]
			Osteogenesis imperfecta, types I–IV	CRISPR-Cas9-mediated HDR editing [36–39]	[35]
Type II (fibrillar)	chondrocytes	*COL2A1*	Type II Collagenopathies		[91]
			Stickler syndrome type I		[92]
Type III (fibrillar)	fibroblasts, smooth muscle cells	*COL3A1*	Ehlers-Danlos syndrome (vascular)		[40, 93]
Type IV (network-forming)	endothelial cells, epithelial cells, podocytes, Schwann cells, smooth muscle cells	*COL4A1*	COL4A1- related disorders		[10]
			HANAC syndrome		[42]
		*COL4A2*	COL4A2- related disorders		[10]
		*COL4A3*, *COL4A4*	Alport syndrome	CRISPR-Cas9-mediated HDR editing [46]	[43, 47]
			Focal Segmental Glomerulosclerosis		[44]
			Thin Basement Membrane Nephropathy		[45]
		*COL4A5*	X-linked Alport syndrome		[43, 47]
		*COL4A6*			[47, 94]
Type V (fibrillar)	mesenchymal cells, Schwann cells	*COL5A1, COL5A2*	Ehlers-Danlos syndrome, (classical)		[40]
Type VI (network-forming)	fibroblasts, chondrocytes, myocytes	*COL6A1, COL6A2, COL6A3*	Ullrich congenital muscular dystrophy, Bethlem myopathy	CRISPR/Cas9-based silencing [49, 50]; exon skipping [51]; GAPMER [52]	[21, 50]
Type VII (anchoring fibril)	keratinocytes	*COL7A1*	Dystrophic epidermolysis bullosa	gene replacement [26, 33, 34, 55, 56]; CRISPR-Cas9-mediated HDR editing [57, 58]; BE/PE [59–62]	[54]
Type VIII (network-forming)	vascular smooth muscle cells	*COL8A1*, *COL8A2*	Fuchs endothelial corneal dystrophy		[95]
Type IX (FACIT)	chondrocytes	*COL9A1*	Stickler syndrome, type IV		[92, 96, 97]
			Multiple Epiphyseal Dysplasia Type 6		[98, 99]
		*COL9A2*	Stickler syndrome, type V		[96, 97]
			Multiple Epiphyseal Dysplasia Type 2		[98]
		*COL9A3*	Stickler syndrome, type VI		[96, 97]
			Multiple Epiphyseal Dysplasia Type 3		[98]
Type X (network-forming)	chondrocytes	*COL10A1*	Schmid metaphyseal chondrodysplasia		[100]
Type XI (fibrillar)	chondrocytes	*COL11A1*	Stickler syndrome, type II		[92]
		*COL11A2*	Stickler syndrome, type III		[92]
Type XII (FACIT)	fibroblasts, osteoblasts	*COL12A1*	Ehlers- Danlos syndrome (myopathic)		[101]
			Bethlem myopathy		[101]
			Ullrich congenital muscular dystrophy		[101]
Type XIII (MACIT)	fibroblasts, myocytes, neurons	*COL13A1*	Myasthenic syndrome		[102]
Type XVII	keratinocytes	*COL17A1*	Junctional epidermolysis bullosa	CRISPR-Cas9-mediated HDR editing [63]	[63]
			Epithelial Recurrent Erosion Dystrophy		[103]
Type XVIII	endothelial cells, hepatocytes	*COL18A1*	Knobloch syndrome, type I		[104]
Type XXV	neurons, myoblasts,	*COL25A1*	Arthrogryposis multiplex congenita		[105]
Type XXVII	chondrocytes	*COL27A1*	Steel syndrome		[106]

To date, no specific genetic disorders have been directly linked to pathogenic variants in the genes encoding type XIV, XV, XVI, XIX, XX, XXI, XXII, XXIII, XXIV, and XXVI collagens.

## Gene editing approaches for collagen diseases

Gene editing shows promise as an innovative approach in modern medicine, offering potential treatments for a wide range of genetic disorders. CRISPR-Cas systems, particularly CRISPR-Cas9, have emerged as the most versatile and widely adopted gene-editing technology [27, 28]. The simplicity, efficiency, and adaptability of CRISPR-Cas technology have propelled its use in diverse applications, from basic research to clinical therapies. CRISPR-Cas9 induces double-strand breaks (DSBs) via the enzymatic activity of the Cas9 endonuclease, guided by a sequence-specific single-guide RNA (sgRNA) [29]. This process occurs when the Cas9 protein, directed by the sgRNA, recognises and binds to a specific DNA sequence adjacent to a protospacer adjacent motif (PAM). To facilitate precise gene editing, the DSBs can be repaired through homology-directed repair (HDR) that utilises a donor template. Concerns regarding the potential risks of DSBs [29] have led to the development of alternative gene-editing strategies, such as BE [30] and PE [31].

These are precise genome-editing technologies that enable single-nucleotide modification without the necessity of introducing DSBs or requiring a DNA donor template [32]. BE uses deaminase enzymes fused to Cas9 nickase to directly convert one base to another, while PE employs an engineered reverse transcriptase to write new genetic information into a specified DNA site [30–32].

While CRISPR-based approaches have garnered significant attention, alternative gene therapy strategies remain crucial. For example, gene inactivation, where the expression of mutated genes is suppressed, and gene replacement therapy, which involves introducing functional copies of genes to compensate for mutated or missing ones [33, 34].

### Type I collagen disorders

Type I collagen disorders are genetic conditions resulting from pathogenic variants in the *COL1A1* and *COL1A2* genes, which encode the α1 and α2 chains of type I collagen. These disorders, including Osteogenesis Imperfecta (OI), some forms of Ehlers-Danlos Syndrome, and Caffey Disease, affect tissues rich in type I collagen, such as bones, skin, and blood vessels, leading to a range of symptoms from fragile bones to skin hyperextensibility [35–41].

**Fig. 3:**
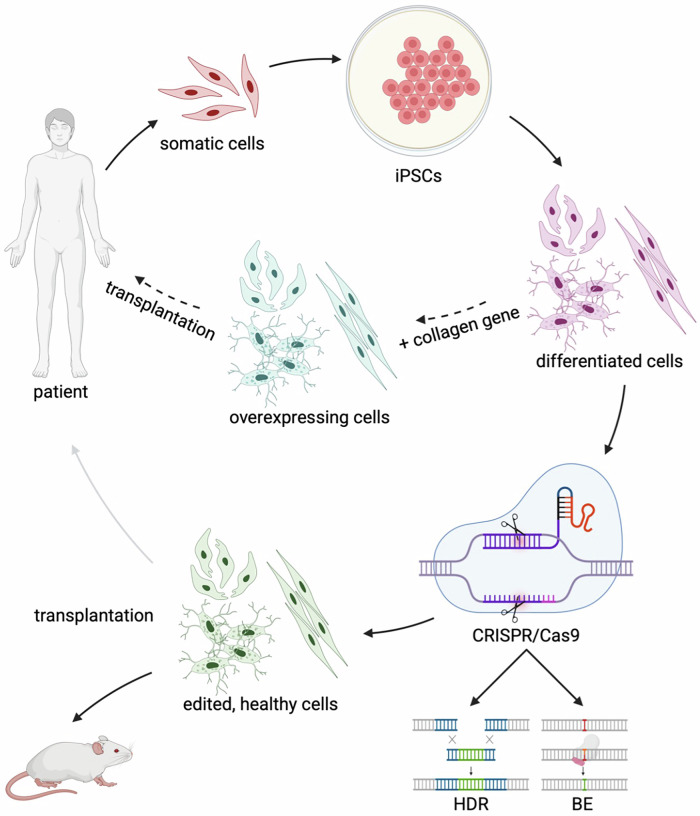
Overview of various CRISPR-Cas9-mediated gene editing approaches. The black arrow represents either homology-directed repair (HDR) or base editing (BE) approaches used to correct somatic cells or iPSC-derived differentiated cells, resulting in gene correction at various levels and modified collagen production. Specifically, HDR has been applied to correct genes, such as COL1A1, COL4A3, COL6A1, and COL17A1. Successfully edited COL7A1 pathogenic variants in dystrophic epidermolysis bullosa (DEB) cells have been transplanted onto mice. Additionally, BE has been tested to correct COL7A1 pathogenic variants in patient-derived fibroblasts. The dashed arrow indicates an ex vivo gene augmentation strategy for recessive dystrophic epidermolysis bullosa, in which samples are transduced with a retrovirus carrying full-length human COL7A1 and subsequently transplanted onto the patients’ wounds. Finally, the grey arrow indicates future ex vivo base/prime-edited cells that could be transplanted back into patients.

OI is a genetic disorder mainly caused by autosomal dominant mutations in the genes that encode type I collagen (*COL1A1* and *COL1A2*). The skeletal and connective tissues are affected, and the disease is characterised by brittle bones, low bone mass, and bone deformities [35]. CRISPR-Cas9-mediated HDR is being explored as a treatment for this disease, both in vitro and in vivo, along with the evaluation of various delivery methods (Fig. 3).

To model the disease, patient-derived induced pluripotent stem cells (OI-iPSCs) were differentiated into osteoblasts, revealing reduced type I collagen production and impaired osteogenic differentiation [36].

CRISPR/Cas9-mediated correction of the *COL1A1* gene in transfected osteoblasts resulted in type I collagen expression, and osteogenic differentiation improved significantly [36]. Similarly, HDR editing was successful (84% editing success rate) when delivered by star polymers (STAR) and restored osteogenic differentiation [37]. Moreover, a unique cis-double-variant c.[175 C > T; 187 T > A] in the *COL1A1* gene was also restored by CRISPR-Cas9-based HDR editing in patient-derived iPSCs, as indicated by increased levels of *COL1A1*, *COL1A2* genes and COL1A1 protein [38].

HDR-mediated gene editing was also tested in vivo in an OI mouse model. A recombinant AAV (rAAV) was used to deliver a CRISPR/Cas9 system containing a partial *COL1A2* DNA template directly to the bone-forming osteoblast lineage cells in the skeleton of one-month-old mice. Treatment improved bone architecture and strength, reduced spontaneous fractures and skeletal deformities, and enhanced overall bone health in the treated mice [39].

The success of CRISPR/Cas9-mediated gene editing in correcting *COL1A1* pathogenic variants for OI serves as a valuable proof of concept for treating other collagen-related disorders and genetic bone diseases, highlighting the broader potential of precision gene therapy in regenerative medicine.

### Type IV collagen disorders

Type IV collagen disorders are a group of genetic conditions affecting the structure and function of BMs throughout the body, particularly in the brain, kidneys and eyes. These disorders result from pathogenic variants in the genes encoding the six α-chains of type IV collagen (COL4A1-6) [10, 42–47].

The most well-known type IV collagen disorder is Alport syndrome, which is characterised by progressive kidney disease, hearing loss, and eye abnormalities. As the disease advances, there is a loss of kidney function, oedema, high blood pressure, hearing loss, and abnormal ocular shaping and colouration [47]. X-linked Alport syndrome (XLAS) is the most common form, accounting for approximately 80% of cases, and is caused by pathogenic variants in the *COL4A5* gene on the X chromosome. Autosomal recessive Alport syndrome (ARAS) is the second most prevalent type, resulting from pathogenic variants in both copies of either the *COL4A3* or *COL4A4* genes on chromosome 2. The rarest form is autosomal dominant Alport syndrome (ADAS), which accounts for about 5% of cases and can be caused by a single inherited copy of a mutated *COL4A3* or *COL4A4* gene [47]. On the side of the spectrum, heterozygous COL4 mutations are also considered a major cause of a pathology that has been referred to as thin basement membrane nephropathy, a pathology estimated to affect as much as 1% of the world population.

A dual-plasmid CRISPR-Cas9 system has been developed to correct pathogenic variants in the *COL4A3* and *COL4A5* genes associated with Alport syndrome [46]. This system included two key components: a plasmid encoding SpCas9 and the guide RNA (gRNA), along with another plasmid that carries the donor DNA template and a mCherry/GFP reporter system to track the activity of Cas9 in cells. To test this system, urine-derived podocyte lineage cells from two Alport syndrome patients were used as disease models. These cells harboured specific pathogenic variants: p.(Gly856Glu) in *COL4A3* and p.(Gly624Asp) in *COL4A5*. The cells were transfected using electroporation, and the results were promising, with correction efficiencies of 44% for *COL4A3* and 59% for *COL4A5*. Importantly, the system demonstrated low rates of unintended indel formation: 10.4% for *COL4A3* and 8.8% for *COL4A5* [46] (Fig. 3).

These results indicate that this dual-plasmid CRISPR-Cas9-mediated editing is effective in correcting pathogenic variants associated with Alport syndrome, achieving high efficiency and relatively low off-target effects. This demonstrates its potential as a therapeutic strategy for other monogenic disorders.

### Type VI collagen disorders

The *COL6A1*, *COL6A2*, and *COL6A3* genes encode type VI collagen, which is essential for muscle tissue integrity [21]. Pathogenic variants in these genes result in a spectrum of disorders ranging from mild Bethlem myopathy to severe Ullrich congenital muscular dystrophy (UCMD). UCMD presents with hyperlaxity of distal joints, contractures of proximal joints, scoliosis and respiratory muscle weakness. At birth, children may present with hypotonia (low muscle tone) and reduced movement [48].

Recent preclinical studies have focused on CRISPR/Cas9-based methods to address the dominant-negative pathogenic variants in the genes encoding type VI collagen.

A CRISPR/Cas9 mediated approach was used to knock out the mutant *COL6A1* allele, specifically targeting the p.Gly275_Lys280del variant, resulting in a significant downregulation of the mutated transcript [49]. Patient-derived fibroblasts were successfully edited with a 32% efficiency in the mutant allele while preserving the wild-type sequence. The edited cells restored collagen VI production and improved the ECM structure [49], highlighting the therapeutic potential of this strategy.

Another study used CRISPR/Cas9-mediated gene editing to silence or correct the COL6A1 c.877 G > A pathogenic variant in dermal fibroblasts [50]. While the correction of the mutant allele through homologous-directed repair occurred at a frequency of less than 1%, measured by next-generation sequencing (NGS) (Fig. 3), frameshift variants led to the silencing of the mutant allele in over 40% of reads without affecting the wild-type allele, confirmed by droplet digital PCR [50].

Additionally, alternative approaches, such as exon skipping [51] and GAPMER technology [52], have shown promise. For instance, the exon skipping of exon 16 of *COL6A3* was investigated using small interfering RNA (siRNA) oligonucleotides in UCMD-derived dermal fibroblasts. These siRNAs selectively suppressed mutant protein expression and improved the quantity and quality of the collagen VI matrix [51]. Gene silencing using GAPMER technology demonstrated the potential for allele-specific silencing. Antisense oligonucleotides (ASOs) targeting an 18-nucleotide heterozygous deletion in exon 15 of *COL6A3* suppressed mutant transcript expression and increased collagen VI deposition in the ECM [52].

These preclinical studies provide promising avenues for developing treatments for collagen VI-related disorders, although further research is needed to translate these findings into clinical applications. The insights may also benefit other collagen disorders by demonstrating the potential of gene editing and silencing techniques to target specific pathogenic variants.

### Type VII collagen disorders

Type VII collagen, encoded by the *COL7A1* gene, is a crucial structural component of anchoring fibrils, which play a vital role in maintaining the integrity of the skin [53]. Pathogenic variants in the *COL7A1* gene can lead to dystrophic epidermolysis bullosa (DEB), a severe skin disorder [53]. Symptoms include widespread skin blistering, ulcers, scarring, dental issues, nail deformity or loss, and an increased risk of skin cancer [54].

Several gene therapy approaches are available to correct pathogenic variants in *COL7A1*, including gene replacement therapy. A lentiviral vector containing the *COL7A1* gene, when injected into the dorsal skin of athymic hairless, immunodeficient mice, resulted in collagen production and deposition in the BM for up to three months [33]. Moreover, the histological defects associated with DEB (including subepidermal blistering, type VII collagen deposition and anchoring fibrils at the basement membrane) were effectively corrected [33].

Based on the early success of gene replacement therapy, two distinct approaches were tested in clinical trials. Beginning in 2010 (NCT01263379), keratinocytes isolated from biopsy samples were transduced with a retrovirus carrying full-length human *COL7A1* and assembled into epidermal sheet grafts, which were subsequently transplanted onto the patients’ wounds **(**Fig. 3**)**. Long-term improvements in wound healing were observed (at 7 years, 70% of treated wounds demonstrated over 50% wound healing compared to baseline). No adverse effects or autoimmunity against type VII collagen were reported [55, 56].

Another gene therapy approach for DEB has undergone testing in a clinical trial that began in 2015 (NCT02493816). The participant received three intradermal injections of a self-inactivating lentiviral vector carrying full-length, codon-optimised *COL7A1* cDNA [34]. The treatment was well tolerated, exhibiting robust and sustained expression of type VII collagen at the dermal-epidermal junction (DEJ) and improved wound healing. Type 7 collagen levels were sufficient to form functioning anchoring fibrils, essential to tissue integrity. Importantly, there were no significant adverse events or immune reactions upon treatment, and lentiviral vectors showed no sign of insertional mutagenesis or off-target effects, highlighting the safety profile of lentiviral vectors for gene delivery in humans [34].

In May 2023, the FDA approved Vyjuvek, a novel gene therapy for DEB [26]. Developed by Krystal Biotech, this topical gel uses a modified herpes simplex virus (HSV-1) to deliver functional *COL7A1* genes directly to skin wounds, enabling cells to produce essential collagen VII protein [26]. Clinical trials showed that 65% of treated wounds closed completely at 24 weeks, compared to 26% with a placebo. The treatment demonstrated a favourable safety profile with minimal side effects, marking a significant advancement in addressing the genetic cause of DEB and offering new hope for patients [26].

Precision gene correction of *COL7A1* pathogenic variants in primary recessive DEB (RDEB) keratinocytes has been achieved pre-clinically using HDR via CRISPR/Cas9 and an AAV-delivered donor DNA template. The approach effectively modified *COL7A1* in cord blood-derived CD34+ cells and mesenchymal stem cells [57].

Similarly, ex vivo HDR was achieved in a variety of *COL7A1* pathogenic variants in RDEB cells and 3D skin-equivalents for the correction of pathogenic variants in exon 2 (c.189delG; p.Leu64Trpfs40) [58]. Through lentiviral delivery, significant cleavage activity was achieved in RDEB keratinocytes and fibroblasts (11% and 15.7%, respectively). Engraftment of corrected 3D skin equivalents onto nude mice rescued expression up to 26%, with normalisation of type VII collagen localisation and anchoring fibril formation at the DEJ [58].

A study of 36 Korean RDEB patients identified 69 pathogenic mutations, with the majority being point mutations (72.5%). Adenine base editors (ABEs) and PE were tested to correct *COL7A1* pathogenic variants in patient-derived fibroblasts. The transplantation of edited skin equivalents into immunodeficient mice resulted in collagen VII deposition and anchoring fibril formation at the DEJ [59]. These findings suggest that base and PE could be viable ex vivo gene editing strategies for RDEB treatment.

In another study, both cytosine base editor (CBE) and adenine base editor (ABE) have shown promising results in correcting pathogenic variants associated with RDEB in patient-derived cells [60]. Evaluation of the CBE3 to correct the c.425 A > G splice-site mutation in *COL7A1* was performed in patient-derived fibroblasts iPSCs. The BE approach achieved c.425 G > A on-target conversion rates of 61% and 45% in fibroblasts and iPSCs, respectively, as confirmed by NGS [60]. While bystander C > T edits at position c.426 were detected, off-target analysis revealed minimal unintended editing. Importantly, the CBE3-mediated correction restored type VII collagen protein expression and secretion in base-edited fibroblasts, as confirmed by IF and western blot analyses. This led to the formation of functioning anchoring fibrils in vivo. Although the number of anchoring fibrils was lower compared to wild-type controls, the quantity was sufficient to restore functional correction of the DEJ, demonstrating the therapeutic potential of BE for RDEB [60].

In a separate study, the adenine base editor ABE8e showed even more impressive results when used to correct a different RDEB-associated pathogenic variant [61]. Electroporation of ABE8e mRNA into RDEB patient-derived fibroblasts resulted in a remarkable 94.6% correction of the pathogenic *COL7A1* gene variant c.5047 C > T (p.Arg1683Ter). This correction restored type VII collagen mRNA and protein expression, as evidenced by western blot and histology of 3D skin equivalents. Notably, ABE8e outperformed its predecessor, ABE7.10, which only corrected 12.4% of mutated alleles [61].

Nanoneedles are an innovative and ground-breaking technology that enable the precise delivery of therapeutic molecules directly into cultured cells (in vitro) or organs (in vivo). This technique offers a safer and more efficient alternative to conventional methods while opening new frontiers in gene editing and regenerative medicine [62].

This delivery method was utilised to deliver an ABE8e plasmid to correct the c.5047 C > T variant in the *COL7A1* gene. A 100% correction efficiency was achieved in RDEB fibroblasts measured by Sanger sequencing, restoring wild-type guanine and preventing the promotion of a premature stop codon [62]. The expression level of type VII collagen protein reached 63.5% of healthy controls, as measured by western blot, with enhanced secretion into the ECM. Nanoneedles demonstrated a strong safety profile with no significant off-target editing events at any of the predicted sites, although a bystander edit at c.5052 resulted in a silent variant (TTA > TTG) with no functional impact [62].

In summary, these diverse gene therapy approaches for DEB demonstrate significant progress in addressing the underlying genetic cause of the disease. Each method offers unique advantages, from the non-invasive nature of topical treatments to the long-term correction potential of lentiviral vectors and the precision of BE. Collectively, these advancements provide a multifaceted approach to treating DEB, offering hope for improved quality of life and potentially curative options for patients with this debilitating genetic disorder.

### Type XVII collagen disorders

Similar to DEB, junctional epidermolysis bullosa (JEB) can result from pathogenic variants in the *COL17A1* gene, which encodes type XVII collagen. JEB arises from pathogenic variants that impact the lamina lucida of the basement membrane zone, causing severe blistering, infections, and potentially life-threatening complications from birth [63].

CRISPR/Cas9 gene-editing techniques have been explored in preclinical studies to correct a frameshift mutation in the *COL17A1* gene, which causes type XVII collagen deficiency in keratinocytes. The specific pathogenic variant targeted was a 2-base pair deletion (c.3899_3900delCT) in exon 52 [63].

Two approaches were investigated: Cas9 nucleases and Cas9 nickases. Both methods were employed in combination with a single-stranded HDR template to correct the pathogenic variant. NGS revealed that when a high-fidelity Cas9 nuclease was employed, an HDR efficiency of 38% was achieved [63]. Notably, utilising Cas9 nickases outperformed the Cas9 nuclease method, restoring ~60% of type XVII collagen expression (Fig. 3). The gene-corrected cells demonstrated significant improvements in cell adhesion and deposition of type XVII collagen along the basement membrane [63].

These results underscore the therapeutic potential of CRISPR/Cas9-mediated gene editing for treating JEB and potentially other genetic skin disorders.

## Current challenges

CRISPR/Cas9-based gene editing for collagen disorders faces several significant challenges, ranging from low efficiency of HDR to delivery system limitations and off-target effects.

The low efficiency of HDR is a major problem with attempts to correct pathogenic variants. For example, in vitro, the CRISPR/Cas9-based gene silencing of the mutant *COL6A1* resulted in correction frequencies below 1%, as detected by NGS to assess the allelic variability [50]. However, more than 40% of reads indicated the silencing of the mutant allele, with a recovery in the collagen VI ECM [50]. This underscores the potential utility of alternative repair mechanisms, such as non-homologous end joining (NHEJ)-mediated gene disruption for dominant-negative pathogenic variants.

To enhance safety, strategies that promote DSB resolution through HDR are vital for facilitating the clinical transition of HDR-based editing strategies. A novel CRISPR platform combines Cas9 with DNA repair factors to block error-prone NHEJ while boosting HDR, enabling precise DNA break repair. This system achieved 7-fold more error-free edits than standard CRISPR/Cas9 across cell lines (HEK293T) and primary human cells (PBMCs) [64].

To further improve the efficacy of HDR, the delivery of DNA templates can be optimised using adeno-associated viral vectors. For instance, an ex vivo HDR gene editing approach applied to *COL7A1* mutant keratinocytes resulted in 60–80% of accurately corrected transcripts [57]. Strategies, such as using single-stranded oligodeoxynucleotides (ssODNs) instead of double-stranded DNA templates and synchronising cells in the S/G2 phase could further improve HDR outcomes [65]. Chemical inhibitors of NHEJ components like DNA-PKcs may redirect repair pathways toward HDR [65].

Furthermore, HDR is associated with off-target effects, resulting in unintended DNA cleavage. Therefore, novel CRISPR-based technologies, like base or PE, might be beneficial [30, 31]. These techniques enable more precise single-nucleotide editing without inducing DSBs, thereby reducing the risk of insertions, deletions, and chromosomal rearrangements [66]. However, bystander edits still remain a significant challenge within the BE systems due to the wide editing window [67], which highlights the necessity of developing more specific and diverse PAM sequences using engineered Cas9 variants or utilising various bacterial species. Editing precision can also be enhanced by modifying the single guide RNA. For instance, the bubble hairpin single guide RNAs (BH-sgRNAs), featuring a 5’ hairpin structure with a bubble region, effectively enhance the specificity of both cytosine and ABEs, reduce off-target editing and maintain on-target efficiency [68]. Moreover, the transient delivery of BE components as ribonucleoproteins (RNPs) could minimise prolonged expression and reduce unintended edits [66].

A suitable in vivo delivery system is the AAVs, but BEs enzymes are too large to be packaged in a single AAV (capacity: ~4.7 kb single-stranded DNA). Consequently, two AAV vectors are typically used, and the intein fragments are reassembled by trans-splicing. Nevertheless, this system is not optimal yet, and more compact BEs have been developed to enable efficient editing at lower doses [67]. These single-vector AAV-encoded BEs can accelerate research by simplifying clinical-scale production and characterisation while reducing AAV-associated toxicity.

An alternative to gene correction is allele-specific gene silencing, particularly for dominant-negative disorders. This approach selectively suppresses mutant alleles while preserving wild-type function. For instance, shRNA-mediated silencing reduced mutant allele expression by 60% in cardiac channelopathies without affecting wild-type alleles [69].

In collagen-related disorders, allele-specific silencing may alleviate disease symptoms by reducing the toxic effects of mutant proteins. For example, the silencing of the mutant allele in *COL6A1* was found in 40% of the reads, with no impact on the wild-type allele [50].

This method can be effective for silencing large genes that may be difficult to edit using BE or PE techniques, but it is crucial to design highly specific guide oligos that can distinguish between mutant and wild-type alleles.

Efficient in vivo delivery of CRISPR-Cas9 components to specific cell types, such as neurons or retinal cells, remains a significant challenge in gene editing applications [70]. The systemic administration of these reagents can result in heterogeneous editing outcomes across different cells, potentially leading to inconsistent therapeutic effects [71]. To address these limitations, AAV serotypes and tissue-specific nanoparticles have been researched to enhance the precision and efficiency of in vivo CRISPR-Cas9 delivery.

As of now, 13 natural AAV serotypes have been identified, but novel hybrid vectors have been developed to improve transduction and limit their tropism to specific cells or tissues [72]. For example, the AAV vector capsid was recently modified by incorporating an endothelial targeting peptide to enhance the efficiency of endothelial-directed gene transfer [73].

Nanoparticles can also be programmed as delivery systems specific to cells and tissues without limited packaging capacity. For instance, collagen IV-targeted nanoparticles, which have been used to release anti-inflammatory agents for vascular diseases gradually [74], could be utilised in various type IV collagen disorders (e.g., Alport syndrome, COL4A1/A2-related disorders) to deliver the healthy *COL4A1-6* DNA as a form of gene replacement therapy.

In addition to nanoparticles, there is another innovative category of non-viral carriers, the RNP complexes. RNPs can deliver preassembled CRISPR-Cas9 protein and sgRNA for transient, DNA-free genome editing, bypassing viral vectors. Their rapid degradation minimises off-target effects and avoids genomic integration risks, enabling precise ex vivo and in vivo applications [75]. Efficient CRISPR/Cas9 HDR-mediated COL7A1 editing was achieved in primary RDEB keratinocytes/fibroblasts (c.6508 C > T). Three sgRNAs targeting the variant were delivered as RNP complexes, and one of the three sgRNAs achieved 73% cleavage efficiency in both cell types [76].

Another novel class of non-viral carriers is the highly branched poly(β-amino ester)s (HPAEs) for delivering plasmids [77]. HPAEs demonstrate robust transfection efficiency (e.g., comparable to Lipofectamine 3000 and jetPEI), resulting in negligible cytotoxicity. For example, CRISPR-Cas9 plasmids delivered via PTTA-DATOD (a type of HPAE) successfully facilitated targeted deletion and reinstated type VII collagen production in keratinocytes from RDEB patients [77].

These advanced delivery methods aim to improve the specificity and consistency of gene editing in target tissues, potentially enhancing the therapeutic efficacy of CRISPR-based interventions.

## Novel approaches

**Fig. 4:**
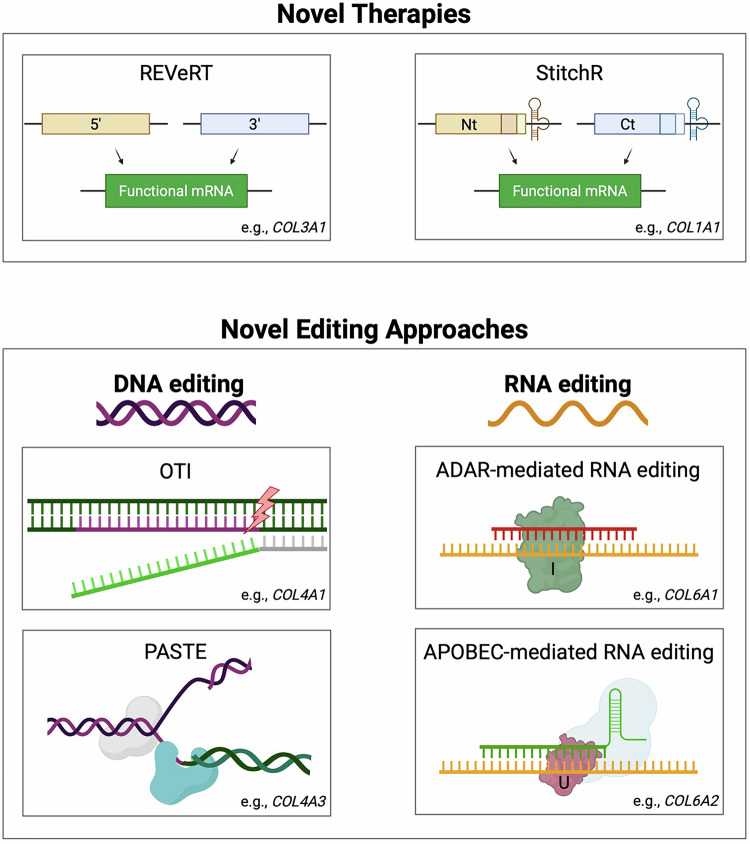
Summary of novel gene therapy and editing techniques. The top panel illustrates two novel trans-ligation therapies: REVeRT and StitchR, offering a one-step approach for whole-gene replacement without causing double-strand DNA breaks. The bottom panel highlights novel editing approaches, divided into DNA and RNA editing. Novel DNA editing techniques include OTI, a sequence-specific and protein-free method, and PASTE, a CRISPR-based genome editing technique that utilises a Cas9 nickase linked to a reverse transcriptase and a serine integrase for inserting extensive DNA sequences. RNA editing approaches feature ADAR-mediated and APOBEC-mediated RNA editing, which introduce targeted modifications to RNA transcripts.

Recent advancements in gene therapy and editing have introduced various innovative therapeutic modalities that offer increased precision and efficiency compared to current methods for treating genetic disorders. Emerging technologies show promising potential for the future of gene therapy: mRNA trans-ligation [78], large DNA sequence integration [79], oligonucleotide-recognising topoisomerase inhibitors (OTIs) [80], and RNA editing via ADAR-recruiting ASOs [81–87]. These innovative approaches can address both collagen disorders and a broad spectrum of other genetic conditions, offering new avenues for treatment (Fig. 4).

Messenger RNA trans-ligation has emerged as a promising strategy to overcome the packaging limitations of viral vectors, such as AAVs, which are constrained to ~4.7 kb [88]. This approach enables reconstitution of full-length mRNA transcripts from smaller fragments, bypassing the need to deliver oversized genes [9] in a single payload.

Building on this, REVeRT (Reconstitution via mRNA trans-splicing) technology splits a target gene into two fragments, packages them into separate AAV vectors, and leverages endogenous splicing mechanisms to seamlessly ligate the split mRNAs post-delivery. This restores full-length protein expression with high efficiency in diverse models, including in vivo applications [89].

Another trans-ligation that enables the precise reconstitution of large therapeutic genes is StitchR [78]. The technique employs specific ribozyme pairs, such as hammerhead or hepatitis delta virus ribozymes, flanking each mRNA fragment. These self-cleaving ribozymes excise their attached RNA sequences post-transcriptionally, generating blunt-ended fragments that ligate without requiring overlapping sequences. Optimising StitchR in mammalian cells led to a remarkable ~900-fold increase in protein expression. Recent preclinical studies have demonstrated the effectiveness of StitchR in treating muscular dystrophies using a dual AAV system to introduce a ~ 6 kb dysferlin or a truncated form of dystrophin (ΔH2-R15). While the study by Lindley et al. [78] showed high efficiency in mRNA trans-ligation, it acknowledged that the process might not be 100% efficient, with some un-ligated transcripts potentially persisting. However, these residual transcripts may either degrade naturally or eventually undergo trans-ligation, highlighting the robustness of this innovative approach in gene therapy [78].

These techniques can potentially be applied to various collagen-related disorders, as the large size of collagen gene coding sequences (typically ranging from 4 to 9 kb) exceeds the packaging capacity of AAVs (4.7 kb). The REVeRT or StitchR-mediated mRNA trans-ligation approaches offer a solution to this limitation by enabling the delivery of functional transcripts, thereby restoring collagen expression and addressing the underlying molecular defects in these disorders.

Programmable Addition via Site-Specific Targeting Elements (PASTE) represents an advanced gene editing strategy that addresses the limitations of traditional approaches for large DNA insertions [79]. This innovative method enables the efficient integration of extensive genetic sequences without introducing potentially harmful DSBs [79].

Unlike conventional knock-in approaches relying on HDR or transposase-mediated insertion, PASTE combines Cas9 (or Cas12k) with serine integrases and recombination landing sites for highly accurate DNA insertion. The mechanism involves Cas9 introducing a targeted single-strand nick at the desired genomic location, followed by serine integrases facilitating the insertion of donor DNA through pre-defined attachment sites (attB and attP) [79]. This site-specific recombination system offers greater control over the insertion process compared to transposases, which can result in random insertions and potential genomic disruption. By avoiding DSBs, PASTE minimises the risk of unintended genomic alterations while improving the efficiency of large-scale DNA insertions. This approach is particularly promising for treating genetic disorders that require the replacement or correction of large genetic sequences, such as monogenic diseases, where the size of the affected gene poses a challenge for conventional gene editing methods [79].

With its ability to precisely and efficiently integrate large genetic sequences without causing DSBs, PASTE technology holds significant promise for treating collagen disorders such as Alport syndrome. By enabling the targeted replacement or correction of large collagen genes like *COL4A3*, *COL4A4*, and *COL4A5*, PASTE could restore the production of functional type IV collagen, addressing underlying molecular defects and improving kidney function in affected individuals.

Another novel technology is a class of gene-editing agents that combine the specificity of an oligonucleotide with a topoisomerase inhibitor, referred to as OTIs. These molecules may potentially facilitate site-specific topoisomerase-mediated cleavage through selective DNA sequence recognition, potentially enabling precise genetic modification. The mechanism of action induces single-stranded breaks without the need for the introduction of foreign proteins, as seen in the CRISPR/Cas9-mediated gene editing platforms. By converting etoposide, a non-specific Top2 inhibitor, into a sequence-specific agent, it is possible that this approach could lead to precise gene modulation whilst minimising off-target effects with conventional Top2 inhibitors [80]. This approach uncovers a potential for site-specific DNA cleavage without the need to introduce foreign proteins [90].

OTIs, with their ability to induce precise, site-specific DNA cleavage without the need for foreign proteins, hold significant potential for treating OI. By enabling targeted correction of pathogenic variants in collagen genes, such as *COL1A1* and *COL1A2*, OTIs could restore functional type I collagen production, addressing the underlying genetic defects and improving bone strength and integrity in affected individuals. Additionally, this technology could be applied to *COL4A1*-related disorders, such as small vessel brain disease, porencephaly, and HANAC syndrome, by facilitating precise correction of pathogenic variants in the *COL4A1* gene, thereby restoring basement membrane integrity and reducing vascular fragility.

RNA editing has emerged as a promising alternative to traditional gene editing, offering transient modifications without inducing permanent genomic changes or off-target DNA effects [81, 82]. This reversible approach provides an improved safety profile for loss-of-function mutations, although it requires repeat dosing due to its temporary nature. The key mechanism involves enzymes, such as adenosine deaminases acting on RNA (ADARs), which mediate adenosine-to-inosine (A → I) editing in double-stranded RNA, with inosine being interpreted as guanine by cellular machinery, and apolipoprotein B mRNA editing enzyme, catalytic polypeptide (APOBEC), which mediates cytosine-to-uracil (C → U) editing, with uracil being interpreted as thymine [82–84].

**Fig. 5:**
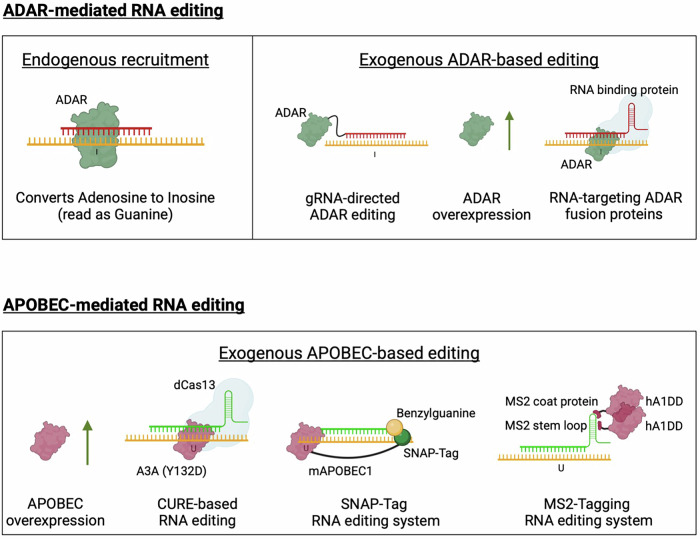
Schematic illustration of ADAR and APOBEC-mediated RNA editing. Endogenous ADAR recruitment (A > I conversions) occurs through a process known as site-directed repair; this mechanism utilises chemically modified ASOs bound to an ADAR-recognising hairpin for recruitment. Exogenous means of ADAR editing involve the overexpression of ADAR, gRNA-directed ADAR editing via tethering exogenous ADAR to a gRNA and RNA-targeting ADAR fusion proteins. APOBEC-mediated RNA editing (C > U conversions) occurs through exogenous means, such as overexpression, CURE-based RNA editing, SNAP-Tag RNA editing, and MS2-Tagging RNA editing systems. Created in Biorender.

RNA editing via ADAR or APOBEC can be facilitated endogenously and/or exogenously (Fig. 5), for example, through the use of ASOs that recruit endogenous ADAR enzymes to catalyse adenosine-to-inosine conversions [82].

Advanced techniques like RESTORE [85] LEAPER [86], and CLUSTER [87] have been developed to achieve precise RNA modifications using ADAR enzymes, employing strategies, such as antisense oligonucleotides, engineered ADAR-recruiting RNAs, and multivalent guide RNAs, respectively [85–87]. Although these approaches show promise, further research is required to address the current limitations and develop safe and effective gene editing therapies for collagen disorders.

RNA editing holds significant potential for treating Ullrich congenital muscular dystrophy (UCMD). By targeting pathogenic variants in collagen genes, such as *COL6A1*, *COL6A2*, and *COL6A3*, which often result in exon skipping or premature stop codons, this approach could correct pathogenic nonsense mutations or splicing defects at the RNA level. Restoring normal type VI collagen production through precise RNA editing offers a promising strategy to address the molecular defects underlying UCMD while avoiding permanent genomic alterations.

## Conclusion

In conclusion, the rapid evolution of gene editing technologies has ushered in a new era of promising therapeutic strategies for a wide range of collagen disorders. Innovative approaches, such as CRISPR-Cas9-mediated HDR, BE, PE, and exon skipping have demonstrated significant potential in both preclinical and clinical studies for conditions including osteogenesis imperfecta, Alport syndrome, and type VI collagen disorders. These cutting-edge techniques aim to address the root cause of these diseases by correcting disease-causing mutations, enhancing collagen production, and ultimately restoring tissue integrity.

The breakthroughs achieved in gene editing strategies for dystrophic epidermolysis bullosa have been particularly noteworthy, establishing a solid foundation for treating monogenic collagen disorders. These advancements have paved the way for developing targeted approaches to address the underlying genetic defects associated with a broader spectrum of collagen-related conditions. The ability to restore a substantial percentage of collagen expression and improve cellular function in preclinical models is especially encouraging, suggesting that these approaches could lead to significant advancements in treating genetic skin conditions and other collagen disorders in the future.

While the potential of these gene editing technologies is immense, it is important to acknowledge that challenges remain. Optimising delivery methods and minimising off-target effects are critical areas that require further research and refinement. Nevertheless, the progress made thus far offers hope for more effective treatments for individuals suffering from these often debilitating conditions.

As we look to the future, continued research and development will be crucial to translate these promising findings into clinically viable therapies. Furthermore, investigating novel gene-editing approaches for other collagen-related diseases will be essential to expand the reach of these revolutionary treatments. With ongoing advancements in the field, gene editing technologies hold the potential to transform the landscape of treatment options for collagen disorders, offering new hope for patients and their families.
